# Middle‐Aged Women With Rett Syndrome: Longitudinal Profile From the British Isles Rett Syndrome Survey and Suggestions for Care

**DOI:** 10.1111/jar.70051

**Published:** 2025-04-07

**Authors:** Anna Hryniewiecka‐Jaworska, Emily Sloper, Hayley Archer, Angus John Clarke

**Affiliations:** ^1^ Institute of Medical Genetics School of Medicine, Cardiff University Cardiff UK; ^2^ All Wales Medical Genomics Service Canolfan Iechyd Genomig Cymru, Cardiff Edge Business Park Cardiff UK

**Keywords:** health care, middle‐aged, natural history, Rett syndrome, social care

## Abstract

**Background and Methods:**

We report historical information from longitudinal data held in the British Isles Rett Syndrome Survey (BIRSS) concerning women of at least 40 years. This information, including comments on the quality of care, has been provided by families, carers, and clinicians.

**Results:**

Information was available on 30 women with a clinical diagnosis of Rett syndrome (RTT), of whom 24 were < 50 years. Twenty‐nine women were diagnosed with classic RTT and one with atypical RTT. Of 18 women tested for *MECP2* mutations, pathogenic variants were identified in 14. There was little increase in severity over time.

**Conclusions:**

The study found that: (1) milder phenotypes were common; (2) depression may be under‐recognised; (3) menopause does not seem to occur early; (4) nutrition standards from the general population will often be inapplicable; (5) multiple opportunities exist to prevent functional decline through detailed attention to the quality of the medical and social care.

**Summary**

- Women over 40 years of age with Rett syndrome.
- Are usually affected by a less severe, nonprogressive form of the condition.
- Are less likely to have had the diagnosis recognised than children with the same condition.
- May experience unrecognised depression.
- Can be actively helped in multiple ways to lead more fulfilling lives if the diagnosis is made.

## 1 Background

Rett syndrome (RTT) is a neurodevelopmental disorder characterised by an apparently healthy first 6 months of life, then stagnation of development and subsequent regression. The child, usually a girl, becomes withdrawn and sometimes distressed, develops stereotypical hand movements and loses skills including purposeful hand use and communication (Hagberg et al. 1983; Neul et al. 2010). She subsequently emerges from regression but remains profoundly affected by dyspraxia, gait ataxia, difficulties with communication and cognition and, perhaps, an element of being “locked‐in”. There is relatively limited information available about adult women with RTT, especially about the natural history of RTT in those above 40 years of age. Accordingly, we have extracted historical data from the now inactive British Isles Rett Syndrome Survey (BIRSS).

Multiple factors are likely to contribute to reduced ascertainment among older women in the British Isles, including (i) the relative lack of familiarity with features of RTT among professionals supporting affected adults, (ii) the lower frequency of referral for diagnostic assessment of adults with cognitive impairment, (iii) the shift in residence from family home to social care as parents age, and a shift from predominantly health care provision to social care, with reduced frequency of health service input when a young person with severe cognitive impairment moves into adult services, and (iv) the often limited access of carers and professionals to information about an adult woman's early childhood. For these reasons, much more is known about children with RTT than about adults. The number of adults reported with RTT is much less than the number of children; population surveys usually give a peak prevalence in mid‐childhood, falling to much lower levels with increasing age.

The higher mortality in affected females compared to the general population will also contribute to this effect (Freilinger et al. 2010; Kirby et al. 2010; Anderson et al. 2014; Tarquinio et al. 2015). The mortality rate in RTT patients in the UK has been estimated at 1.2% per annum (Kerr et al. 1997). This effect is likely to be more marked in those more severely affected, although data may be skewed by possible underdiagnosis in more mildly affected individuals. Some patients develop additional clinical features, such as gastrointestinal problems of likely autonomic origin, which may blur the clinical picture of RTT (Nielsen et al. 2001; Smeets et al. 2003; Roze et al. 2007; Brunetti and Lumsden 2020).

There are few longitudinal studies of women with RTT, and most follow a small number of patients under 40 years of age (Berg and Hagberg 2001; Hagberg et al. 2001; Hagberg et al. 2003; Smeets et al. 2003; Hagberg 2005) and consensus statements acknowledge that further experience with older patients is needed (Fu et al. 2020). The larger natural history studies also concentrate on younger patients (Anderson et al. 2014; Neul et al. 2014; Tarquinio et al. 2017), and those studying adult women generally have few patients over 40 years of age (Tarquinio et al. 2015; Cianfaglione et al. 2016; Bisgaard et al. 2021; Peron et al. 2022). Cross‐sectional data have also been used in studies on larger populations including older people with RTT (Smeets et al. 2003; Cass et al. 2003; Halbach et al. 2008; Smeets et al. 2009; Vignoli et al. 2012; Neul et al. 2024). A review of published experience indicates aspects of care to address (Lotan et al. 2009). The aim of the present study was to report the available longitudinal data concerning 30 middle‐aged survivors known to BIRSS and to note helpful interventions for the health and social care of adults with RTT.

## 2 Methods

Data for 30 women over the age of 40 years, about whom information was gathered on at least two occasions, were ascertained from the BIRSS. The BIRSS was established by Dr. Alison Kerr in Glasgow in 1990 (Kerr et al. 2003) but transferred to Cardiff when Dr. Kerr retired in 2005. The original intention from 2005 was to update the patient records every 5 years, but this was not achieved due to a reduction and subsequent cessation in funding for BIRSS. However, historical data were extracted before the database was discontinued. The data were provided by parents and carers of those affected, collected by postal questionnaire and supplemented by telephone conversations, information from other clinicians, and occasionally in person at the Cardiff RTT clinic.

All patients on whom at least some data were available from > 40 years of age, who met the clinical diagnostic criteria for RTT, and had at least two data collection points for the BIRSS (survey questionnaires) were included in this study, drawing on earlier survey information as well as that from > 40 years. Patients did not require a molecular diagnosis to be included. Patients were classified according to the revised diagnostic criteria for RTT (Neul et al. 2010).

A Simplified Severity Score (SSS) was calculated for all women in the study for each decade of their lives using information in the BIRSS health questionnaire (Table 1). This score, the second, simpler score of the two used by Smeets and colleagues, excludes clinical features not normally present in milder RTT and includes factors considered likely to influence the long‐term evolution and severity of RTT (Smeets et al. 2009). The probable score for the early decades of some patients was deduced from the available information.

**TABLE 1:** Simplified severity score, from Smeets et al. (2009).

Score	0	1	2	3
Sitting	Normal	Impaired	Lost	Never acquired
Walking	Normal	Impaired	Lost	Never acquired
Hand use	Normal	Reduced	Lost	Never acquired
Speech	Normal	Some words	Lost	Never acquired
Epilepsy	No seizures	Controlled	Uncontrolled	Status epilepticus
Spine deformation	Absent	Mild	Severe	Operated

*Note:* Maximum score: 18; score < 9 indicates a milder phenotype.

The data collected also included patient demographics and antenatal, medical, and family history. Feeding difficulties were assessed by calculating a Feeding Difficulty Score, with a maximum score of 8 (Kerr et al. 2006).

## 3 Results

**FIGURE 1:**
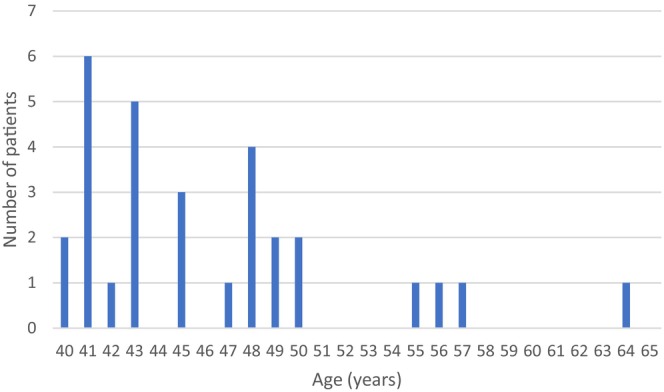
Ages of patients at the time of the last completed survey health questionnaire (years).

Of 30 middle‐aged women with RTT (aged 40 years and over), 29 had Classic RTT and one had atypical RTT. One woman was in her 60s, 5 were in their 50s, and 24 were in their 40s (Figure 1).

### 3.1 Molecular Analysis

Eighteen of the 30 women had *MECP2* analysis, of whom 14 (78%) had identified mutations. Eight women had not been tested and no information was available for the remaining four. Among those with identified *MECP2* mutations, six were missense mutations (one Arg133Cys, two Arg306Cys, one Thr158Met, one Pro255Arg and one Pro133His); one was an early truncating mutation (Arg255X), two were late truncating mutations, three were small, intragenic, C‐terminal deletions, and two were large (exonic) deletions. Those women who did not have a molecular diagnosis instead fulfilled the diagnostic criteria for a clinical diagnosis of RTT. One explanation for the lack of molecular confirmation in some women relates to laboratory practises when their genomic testing was performed. Many were among the first patients tested for RTT in the United Kingdom, at which time the detection rate was lower (approximately 80%), likely due to limitations of the technology (Cheadle et al. 2000). Molecular testing was not repeated in this clinic because women were referred from centres across the UK and abroad. Clinical diagnoses were established in person by Dr. Kerr before transfer of the survey in 2005, or confirmed at the Cardiff RTT clinic, or both.

### 3.2 Severity

**FIGURE 2:**
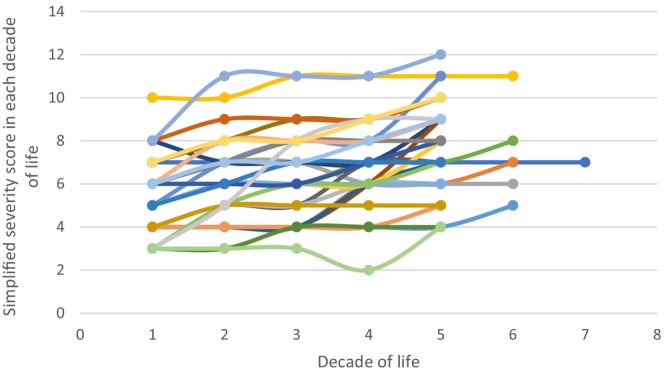
Each patient's ‘simplified severity score’ in each decade of life (Smeets et al. 2009).

Where possible, Smeets's SSS was calculated for each decade of life (Smeets et al. 2009). It remained below 9 for 19 patients in all decades of their lives. When calculated, the SSS for 24 women (80%) was 9 or below, for three women (10%) was 10, and for three women (10%) was 11 or 12. Very little increase in severity was observed over the decades and, even when the severity increased, the score usually remained below 9 (Figure 2).

### 3.3 Gross Motor Skills

Two (7%) women never walked and two (7%) lost the ability to walk during their childhood regression. Of those 26 women walking after their childhood regression, two (8%) permanently lost their ability to walk in their 20s and nine (37.5%) of those still walking in their 40s lost that ability during their 40s or 50s. Of the 14 women known to continue to walk, all had an abnormal gait with a stooped posture on a spectrum of ability from walking independently, although with a mildly ataxic gait, to walking but requiring a variable degree of support. Sitting ability was well preserved: 26 of 30 women could sit in their 40s, 13 requiring support.

Of the women who could walk, contractures were present in 11 (79%), not present in two (14%) and there was no information for one woman. Information was not specifically recorded about the location of the contractures in this cohort. Four women had corrective operations for contractures, including tendon lengthening or tendonotomies. Hip problems (dislocation, displacement, rotation) were reported in 12 women (Table 2).

**TABLE 2:** Summary of gross motor skills.

Gross motor skill		Number of women	%	Comments
Walking	Never walked	2/30	7	
	Lost ability to walk during regression	2/28	7	
	Lost ability to walk during 20s^a^	2/26	8	One in association with other features of regression
	Lost ability to walk during 30s^b^	0/24	0	
	Lost ability to walk during 40s^c^	7/24	29	
	Lost ability to walk during 50s^d^	2/17	12	
Sitting ability	Able to sit after regression	29/30	97	
	Lost ability to sit in 40s	3/29	10	
	Could sit independently in 40s	13/29	45	
	Could sit with support in 40s	13/29	45	

^a^ Those walking through teenage years.

^b^ Those walking through their 20s.

^c^ Those walking through their 30s.

^d^ Those walking through their 40s.

### 3.4 Hand Use

All women could use their hands to some extent before regression. Eleven women (37%) regained hand use and could finger feed throughout their lives. One had a late apparent regression including a loss of her ability to use hand skills in her 20s. Ten women (33%) never regained hand use after regression. Three women lost their ability to finger feed in their 30s, 40s and 50s. One woman regained her ability to finger feed in her twenties and lost it again in her 40s. Another woman regained her finger feeding in her 30s and was still feeding herself in her 40s. Longitudinal information on this was not available for three women but they could not use their hands at one time point: one in her 50's and two in their 40's (Table 3).

**TABLE 3:** Summary of speech skills.

Speech skills		Number of women	%	Comments
Early speech	No data available	1/30	3	Woman in her 60s—no record of early speech
	Never babbled or used words	1/30	3	Diagnosis of atypical rett syndrome
	Able to use words prior to regression	28/30	93	
Later speech	Permanently lost ability to speak after childhood regression	21/28^a^	75	
	Recovered words then lost speech after apparent regression in adulthood	1/28^a^	4	Lost words after having the symptoms of a regression in her 20s
	Others who spoke after childhood regression	4/28^a^	14	Two had words in their 30s and lost them in their 40s and two retained a few simple words, one of whom continued to use phrases of 2–3 words in her 40s.
	Data about regaining speech after regression unavailable	2/28^a^	7	

^a^ Of 28 women who were known to have used words at some stage.

Stereotypies were described in all the women at some point during their lives, especially midline hand movements and hand‐to‐mouth movements. Although the reports were subjective from parents or carers, stereotypies in later life were described as milder, less intense, and sometimes only affecting one hand or manifesting during periods of agitation. In some cases, stereotypies disappeared with age. In several, the ability to use one hand improved when the other hand was held gently.

### 3.5 Speech, Understanding and Communication

Twenty‐eight of the 30 women could use words before regression, with most losing this ability during childhood regression. Of the five women known to recover words after regression, one lost these in her 20s, two in their 30s, and two continued to use words at least into their 40s, one including short phrases.

**FIGURE 3:**
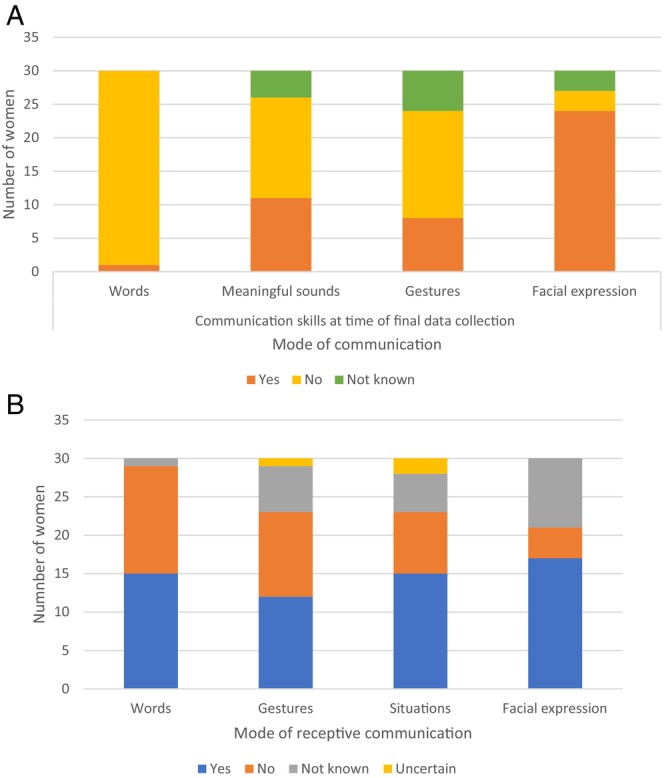
Expressive (A) and receptive (B) communication ability at the final data collection point for all women with RTT.

Information regarding other means of expressive communication was available for 26 of the 30 women. Eleven used meaningful sounds, 24 of the 29 women for whom data was available could use facial expressions, and eight of the 26 women could communicate by gesture (Figure 3A).

Regarding receptive communication, 15 of 29 women could understand words without gestures, 13 of 27 could understand gestures, and 15 of 25 had some understanding in specific contexts. Seventeen of 23 were said to have some appreciation of facial expression (Figure 3B).

With regards to the use of eye contact, 23 women maintained good eye contact, three had poor eye contact, and three had no eye contact. One woman, previously reported to have poor eye contact, regained good eye contact at 62 years after a customised chair was made, which resulted in better positioning of her head.

### 3.6 Epilepsy

Seizures were reported for 18 of 30 women (60%) at some stage during their lives. Ten (33%) had epileptic seizures in middle age, usually generalised tonic–clonic (GTC) or myoclonic seizures, with the seizures being well controlled on medication in nine. Five women had been seizure‐free for many years; two remained on carbamazepine, and medication was being withdrawn or had been stopped in three others.

One woman with a few GTC seizures per year had not taken regular anticonvulsant medication since 27 years of age. In one patient with ongoing seizures, there was difficulty differentiating between autonomic episodes and epilepsy. Similar difficulties had previously been reported for two further women, now seizure‐free.

### 3.7 Scoliosis

All 30 women had scoliosis that progressed slowly throughout their lives, but only one was reported as having undergone surgery. This may be because these decisions were made some decades ago. Eighteen women had mild scoliosis, and four developed scoliosis beyond the fourth decade. Sixteen of the 18 women with mild scoliosis could walk in early adulthood. Twelve women had severe scoliosis, of whom one never walked and required surgical treatment; all had abnormal muscle tone (hypertonia or dystonia). The other lady who never walked had mild scoliosis.

### 3.8 Breathing

Of the 30 women, 27 intermittently hyperventilated and 25 had breath‐holding spells, with or without vacant spells, at some stage. The frequency of hyperventilation episodes decreased in many of the 18 women still affected in middle age; 16 had ongoing vacant spells and they all suffered breath‐holding episodes.

### 3.9 Growth

The majority of the women were of short stature, with their height below the 2nd centile. The median weight was approximately the 2nd centile and ten (one third) were underweight (Table 4); 37% had a normal BMI (Table 5). However, unusually for RTT, a majority (17/28, 61%) had a normal head circumference, above the 2nd centile, as might be expected in this group with a relatively mild phenotype.

**TABLE 4:** Growth parameters for middle‐aged RTT women from (name of survey).

Measure/centile	< 0.4th	0.4th–2nd	> 2nd	Unknown
Height	14	7	7	2
Weight	8	5	14	3
OFC	6	5	17	2

**TABLE 5:** BMI for middle‐aged RTT women from (name of survey).

Weight category	Underweight	Normal	Overweight	Obese	Not known
BMI	< 18.5	18.5–24.9	25–29.9	30 and above	
Number	10	11	4	2	3

### 3.10 Feeding and Nutrition

Data regarding feeding and nutrition were available for 28 patients. Nineteen women had minimal to moderate feeding difficulties (feeding score ≤ 3); nine women had moderate to severe feeding difficulties (feeding score 4–7). Despite this, only three women were fed enterally via tube: two via PEG or PEJ inserted in their fifth decade following aspiration pneumonias, and the third via PEG inserted because of unexplained weight loss in her 30s. Overall, 82% of women in our study had problems with swallowing, chewing, secretions and/or appetite.

Further information was obtained from carers of 22 women with RTT. Two had severe difficulties with maintaining effective mouth closure, two had poor posture affecting feeding, four had difficulty chewing, two had difficulty swallowing, five had excessive secretions, one had poor appetite, and two had difficulty drinking.

### 3.11 Mood, Behaviour and Sleep

Twenty‐eight (93%) of the 30 women currently experience or previously experienced episodes of excitement and/or agitation, including 10 who had episodes of laughing. Nineteen women demonstrated self‐injurious behaviours at some point in their lives. Injurious behaviours toward others were reported in six women; for one, this occurred only when she was pre‐menstrual. Autistic features were described in one lady who did not relate easily to others and lacked emotional warmth to her family, avoiding eye contact, disliking disruption to her daily routine, and preferring repetitive activities such as paper flicking (Table 6).

**TABLE 6:** Descriptions of behaviour experienced by middle aged women with Rett syndrome.

Described behaviour	Number of women (/30)	%
Episodes of excitement and agitation	21	70
Episodes of laughing	10	33
Previous episode(s) of excitement and agitation	7	23
Self‐injurious behaviour at time of assessment	10	33
Self‐injurious behaviour in the past	9	30
Injurious behaviour toward others	6	20
Episodes of unexplained sadness at time of assessment	21	70
Episodes of unexplained sadness previously	4	13
Unexplained weight loss during adulthood	9	30

Twenty‐five women (83%) had episodes of unexplained sadness at some point in their lives. Nine women (30%) had unexplained weight loss during adulthood. For one woman, a temporary period of deterioration was reported with transient loss of mobility and bladder control, cessation of menstruation, and apparent depression in her late twenties.

Although not specifically covered in the BIRSS questionnaire, anxiety (including social phobia and agoraphobia) was reported in comments made about five women, either in clinic or in the free text section of the questionnaire. One woman in her 50s had severe mood swings with agitation, treated with imipramine and lithium. One woman had severe anxiety in her 40s, treated with risperidone. One woman required carbamazepine as a mood stabiliser for severe tantrums. Three women were also treated with fluoxetine for low mood, and one took 5‐hydroxytryptophan.

Grief reactions were described in three women. One woman was described as depressed and ‘incredibly upset and frightened’ after her grandfather's death. Another became withdrawn, losing her appetite and losing weight in reaction to her father's death. Another woman was described as ‘very sad when someone sings a song that her mother used to sing to her before she died 17 years previously. That is the only time she seems to cry’.

73% of women had experienced sleep difficulties at some point in their lives (Table 7). Eight women were not described as having had sleep difficulties, but it is possible that this may reflect recall bias among their carers.

**TABLE 7:** Sleep disturbances reported.

Sleep disturbances	Affected/total	%
Daytime nap requirement	15/30	50
No sleep problems reported during whole life	8/30	27
Prior sleep problems, resolved by middle age	8/30	27
Sleep disruption reported in middle age	14/30	47
New phenomenon in middle age	3/14	21
Improvement noted	11/14	79

### 3.12 Menstrual Status

Data about menstruation status were available for 27 women. Five women had stopped menstruating (aged 40–53 years at the time of report) and one was peri‐menopausal.

### 3.13 General Health

Common symptoms included constipation, requiring regular use of laxatives, joint contractures, and small cold feet. Gastro‐oesophageal reflux, upper and lower respiratory tract infections, aspiration pneumonias, and chronic otitis media were also reported. For two women (one in her twenties and another in her 30s), an acute period of deterioration was reported and was associated with obvious distress, loss of mobility, and, in one case, loss of bladder control and menstruation. No cause was found; the two women recovered although their mobility was impaired thereafter. Diet‐controlled diabetes, kidney stones and urinary tract infections, rheumatoid arthritis, gingivitis, allergy to cats and dogs, asthma, hay fever and eczema, blepharitis and conjunctivitis, rosacea, sebaceous cysts, uterine fibroids, vaginal candidiasis, myopia, and hallux valgus were also reported. Data were not specifically collected on objective measures of osteopenia or osteoporosis, as that was not routinely assessed in women with RTT at the time. Three of the women (10%) had experienced one or more fractures (Table 8).

**TABLE 8:** General health features reported in middle‐aged women with RTT.

Morbidity	Number	Comment
Contractures	23/30	Mostly at knees and ankles
Hip problems	12/30	Dislocation, displacement, rotation
Small cold feet	20/24	
Short 4th metatarsal	8/20	
Constipation	23/30	Required regular laxative use
Fractures	3/30	
Hypothyroidism	2/30	
Hyperthyroidism	1/30	FH of thyroid dysfunction known
Iron deficiency anaemia	1/30	
Acute period of deterioration associated with distress and loss of mobility	2/30	No cause identified, both recovered apart from ongoing impairment to mobility

## 4 Discussion

There was a greater prevalence of milder disease among the older women with RTT in our study, consistent with the findings of others (Smeets et al. 2009; Tarquinio et al. 2015). This indicates a survival advantage for the most mildly affected patients (Kerr and Prescott 2005). Some patients were able to walk, talk, and use their hands at least into early middle age. Some features of RTT improved with aging, for example, epilepsy, agitation, sleep, and hand stereotypies. However, there was late motor deterioration in some, including the development of abnormal muscle tone with loss of walking. Other late‐onset deteriorations included loss of speech, increasing feeding difficulties, and constipation. The potential to recover skills lost during temporary setbacks in adulthood was demonstrated in some women, as with episodes interpreted as resulting from depression or of uncertain origin. Although the use of words was present in only two women, most achieved some communication by other means. In general, our longitudinal data show a substantial stability in severity over the adult years (Figure 2).

Milder phenotypes have been reported to be more likely in association with specific *MECP2* gene mutations or with favourably skewed X‐chromosome inactivation (XCI) (Cheadle et al. 2000; Leonard et al. 2003; Kerr et al. 2006; Smeets et al. 2009; Bebbington et al. 2010; Halbach et al. 2012; Neul et al. 2014). However, caution is recommended in the interpretation of genotype–phenotype relationships, particularly when counselling the parents of newly diagnosed girls, due to the variability in clinical phenotypes. Clinical variability has been documented in natural history studies (Anderson et al. 2014; Neul et al. 2014; Tarquinio et al. 2015; Tarquinio et al. 2017) and our own work has demonstrated the poor correlation between severity and X chromosome inactivation in lymphocytes (Archer et al. 2007). These studies reinforce the conclusion that genotypes are useful in providing explanations for established neurodevelopmental phenotypes but are inadequate for the prediction of how the clinical phenotype may evolve in an individual case (Shahbazian and Zoghbi 2001; Weaving et al. 2003; Archer et al. 2006; Archer et al. 2007; Bebbington et al. 2008; Halbach et al. 2012; Neul et al. 2014). Clinical assessment of the timing and severity of phenotypic manifestations remains the most important prognostic factors (Smeets et al. 2003; Schanen et al. 2004).

### 4.1 Gross Motor

Gross motor activity, performance, and muscle strength generally deteriorate over the years in RTT (Dunn and Macleod 2001; Steffenburg et al. 2001; Hagberg 2002; Kerr 2002; Williamson and Christodoulou 2006; Roze et al. 2007; Halbach et al. 2008; Bisgaard et al. 2021). People with RTT can undergo premature neuromuscular aging, and peripheral atrophy is often seen, usually combined with dystonic‐rigid signs (Hagberg 2005). Increased muscle tone, spasms, and contractures can also be problematic (Roze et al. 2007; Dunn and Macleod 2001). Abnormal muscle tone, posture, and locomotion increase the risk of contractures, malposition, and loss of motor function (Larsson and Witt‐Engerström 2001). Plantar flexion, peroneal weakness, and scoliosis become more prevalent with age (Witt‐Engerstrom 1992). While muscle tone may be reduced earlier in life, it is often increased in adults (Kerr and Stephenson 1985). However, some of those in our study who could walk with little impairment were reported to have near normal muscle tone.

Walking skills may be greater than is commonly assumed, with lack of walking resulting from a lack of opportunity and training [Jacobsen et al. 2001; Schönewolf‐Greulich et al. 2017]. Contractures can also represent a potential barrier to continued walking (Kerr and Burford 2001). Fixed joints become more common with age, with contractures present in 95% of adults in one study (Cass et al. 2003). Many such contractures may be prevented by regular passive exercises through the full range of movement (Kerr 2002). Abnormal muscle tone and posture can contribute to the development of hip displacement in RTT, and screening for this is advised in middle age (Tay et al. 2010).

### 4.2 Hand Use

Loss of purposeful hand movement is usually noted in those with RTT, even when less severe (Kerr 2002; Stallworth et al. 2019). Hand stereotypies are a typical manifestation in RTT and often occur early and persist throughout life (Roze et al. 2007; Vignoli et al. 2009; Stallworth et al. 2019). Reports on the natural history of hand function and description of hand stereotypy in RTT vary (Witt‐Engerström 1990; Cass et al. 2003; Carter et al. 2009; Downs et al. 2010). In adult women, hand stereotypies tend to involve the hands being held apart, whereas in younger girls they are typically held together (Kerr et al. 1987; Cass et al. 2003) suggested it may result from restriction of movement or possibly a lower level of arousal or agitation. Despite stereotypies, many of the women who survived to middle age had some use of their hands, mostly in finger feeding. Regaining this ability in later life has been reported, although it was not seen in our cohort and can easily be lost when not encouraged (Jacobsen et al. 2001; Schönewolf‐Greulich et al. 2017), as seen in one woman reported here, when self feeding was discouraged in residential care.

### 4.3 Speech

Speech was uncommon in the women reported here, as elsewhere (Halbach et al. 2013), although there was substantial ability to communicate. Speech is possible among older women with mild phenotypes, being most probable in those with missense and late truncating mutations including C‐terminal deletions (Nielsen et al. 2001; Zappella et al. 2001; Kerr et al. 2006). Cognitive and communication skills do not appear to decline with age and may improve (Hagberg 2002; Cass et al. 2003; Halbach et al. 2008; Halbach et al. 2013). Eye contact is frequently well‐preserved in adulthood (Hagberg et al. 2001; Hagberg 2005; Halbach et al. 2008; Schönewolf‐Greulich et al. 2017). Women cared for at home are more likely to be reported as communicating effectively (Didden et al. 2010), possibly representing a greater awareness in the families of the women's abilities. Carers of women with RTT, both at home and in residential facilities, should encourage communication and decision making by the women (Schönewolf‐Greulich et al. 2017). This may require attention to appropriate seating and head support to enable eye contact.

It is possible that older women with better quality preserved speech are less likely to have been diagnosed with RTT and are absent from our study for that reason.

### 4.4 Epilepsy

As we also found, epilepsy has previously been reported to be less prevalent in later life, often with complete resolution of seizures (Steffenburg et al. 2001; Hagberg 2002; Hallbach et al. 2013). The differentiation of non‐epileptic vacant spells and epileptic seizures remains problematic in middle‐aged women, and therefore the incidence of epilepsy may be over‐reported in this age group (Julu et al. 2001; Glaze et al. 2010; Tarquinio et al. 2017; Henriksen et al. 2018). Video EEG monitoring could help provide definitive information and avoid the inclusion of other non‐epileptic events such as vacant spells, episodic laughing, crying, or staring (Glaze et al. 1998; Pintaudi et al. 2010). In our study, most middle‐aged patients continued to take anti‐epileptic medications despite having had no seizure for years, consistent with other reports (Cass et al. 2003; Halbach et al. 2013; Tarquinio et al. 2017).

Regular review of anti‐epileptics is important in RTT; withdrawal should be considered in those who have been seizure free for a significant time (Steffenburg et al. 2001; Hagberg 2002; Halbach et al. 2013; Tarquinio et al. 2017). This is especially important because osteoporosis has been reported in RTT (Budden and Gunness 2003; Motil et al. 2008); the prolonged use of anti‐epileptic medications can contribute to this (Leonard et al. 2010).

### 4.5 Osteopenia and Osteoporosis

Only 10% of the women in this report had a history of fractures, but the failure of BIRSS to have collected data on osteopenia or osteoporosis is disappointing. Periodic screening for low bone density should be considered in all adult women with RTT, as osteopenia can be present from a young age (Motil et al. 2008). Other risk factors for osteoporosis frequently present in adult RTT women include a sedentary life, nulliparity, low body weight, prolonged use of depot contraceptives, and inadequate sun exposure, as well as long‐term anti‐epileptic medication [Ryan et al. 2002; Zysman et al. 2006; Motil et al. 2008]. The risk of fractures in RTT is greater than in the general population, and fractures can be missed by carers due to communication difficulties and the apparently high pain threshold of many RTT patients (Downs et al. 2008).

### 4.6 Scoliosis

Scoliosis was present in all women in our study. To prevent progression of scoliosis and improve seated position and walking ability, early surgical intervention is usually recommended (Kerr et al. 2003; Thorey et al. 2007; Downs et al. 2009; Bisgaard et al. 2021). Surgery is most beneficial when the woman is well‐nourished and active (Kerr et al. 2003). In our study, only one woman with severe scoliosis was managed with corrective surgery. Given that the women included in the study were often from other regions or countries, we cannot comment on why the others were not managed surgically.

### 4.7 Breathing

In line with the findings in this study, breathing dysrhythmias such as apnoeas, hyperventilation, and breath‐holding spells are common in RTT but have been shown to decrease in adulthood (Witt‐Engerström 1990; Cass et al. 2003; Halbach et al. 2013; Tarquinio et al. 2018). However, either the failure to initiate inspiration or episodes of prolonged inspiration can persist in many adult women (Witt‐Engerstrom 1992). Valsalva breathing patterns are also characteristic and can lead to a sudden fall in blood pressure, fainting, and abdominal bloating (Kerr et al. 2003; Witt‐Engerstrom 1992).

### 4.8 Growth

Adults with RTT tend to be short (Tarquinio et al. 2012): the average adult height is approximately 136 cm, i.e., 30 cm below the population mean (Holm 1986; Percy 1992). Adults with RTT usually also have small hands and feet, often with shorter fourth digits, especially the metatarsals (Kerr et al. 1995; Kerr 2002).

The proportion of women with RTT who have short stature or are underweight increases with age (Holm 1986; Percy 1992), with the progressive loss of muscle and bone mass (Motil et al. 2008; Smeets et al. 2009). Their BMI may therefore be low despite a normal fat mass, and the use of general population nutrition standards will often be inappropriate. Skin fold measurements or bioelectrical impedance analysis to estimate body composition may be more useful (Letellier et al. 2010). The lower lean body mass of women with RTT contributes to their lower daily energy expenditure (Motil et al. 1998). It is important to tailor nutritional strategies to the specific needs of individual women, who do not always require increased calorie intake but may require supplementation with specific vitamins and minerals, including calcium, Vitamin D, and iron (Zysman et al. 2006; Schwartzman et al. 2008; Tarquinio et al. 2012).

### 4.9 Feeding Difficulties

Feeding difficulties, especially fluid intake, tend to worsen with age because of increased tongue muscle tone (Kerr 2002). Adult women have been reported to have more problems with breathing and are more likely to be constipated, especially if fluid intake is limited; these can both interfere with feeding (Halbach et al. 2008). Gastro‐oesophageal reflux, toothache, oral thrush, change in daily routine, and depression may also be considered when loss of appetite is reported in adult patients (Kerr 2002), but it can arise for no clear reason (Cass et al. 2003).

### 4.10 Mood

After adolescence, mood has been reported as likely to improve (Sansom et al. 1993; Halbach et al. 2013), contrasting with the findings in this study and two previous studies (Cianfaglione et al. 2016; Hryniewiecka‐Jaworska et al. 2016). Depression is not commonly reported (Sansom et al. 1993; Kerr 2002), although it may be under‐reported and therefore under‐treated (Hryniewiecka‐Jaworska et al. 2016). Unexplained changes of appetite, weight loss, sleep disruption, injury to others, fear, restlessness, crying and/or anxiety may be signs of depression or bereavement reactions when no other medical explanation is found. Adult women with RTT may be exposed to numerous stressful events: leaving school, loss of school friendships, change of services, moving away from home and family, and the loss of grandparents, parents, carers or co‐residents, any of which could trigger depressive reactions.

### 4.11 Other Health Problems

Women with RTT may also experience a range of common health problems. However, the health issues found to be more common in people with RTT include undiagnosed depression (Hryniewiecka‐Jaworska et al. 2016), osteoporosis, abnormal thyroid function, and diabetes (Cooke et al. 1995), and iron deficiency anaemia (Schwartzman et al. 2008). In contrast with a previous study of adults with intellectual disability, women in our study did not experience early menopause (Martin et al. 2003).

### 4.12 Limitations

The authors acknowledge that there were limitations to the study, particularly relating to the qualitative nature of the data. The data were collected retrospectively and relied on parent, carer, or clinician recall. It is possible that the SSS of Smeets et al. (2009) did not reliably capture the range of possible symptoms, potentially resulting in the omission of some details pertaining to the women's health or the decline in their health. There was no specific funding available for the study. Therefore, it was not possible to review patients clinically during each decade of their life, particularly given their geographical distribution. Finally, the molecular data available could not be confirmed for every woman and relied on evidence from clinic letters and reports or parental recall. Those without a molecular diagnosis were included on the basis of a clear clinical diagnosis in keeping with the diagnostic criteria, which are purely clinical (Neul et al. 2010). Although unlikely, we acknowledge the possibility of an alternative diagnosis in some patients.

**TABLE 9:** Recommendations for the health and social care of middle‐aged women with RTT.

Theme	Recommendation
Transition to adult care	Care needs to be taken to ensure that young adults with RTT continue to receive multidisciplinary care (Peron et al. 2022)
Genetic testing	Genetic counselling and testing should be offered all with a suspected diagnosis and considered for women with a pre‐existing diagnosis
Mobility	Carers should encourage continued mobility where possible.
	Screening for risk of hip displacement is advisable from middle age.
Hand use	Hand use can sometimes be directly encouraged by gently separating the hands and holding the other hand apart.
Communication	Efforts to encourage communication and decision making should be made.
	Attention should be paid to appropriate seating and head support to enable adequate eye contact.
Feeding and nutrition	Self‐feeding should not be discouraged.
	Consider supplementation with specific vitamins and minerals, including calcium, vitamin D and iron.
	Adequate fluid intake should be encouraged.
	Standard means of measuring nutritional status may be inappropriate and skin fold measurements or other measures may provide a better guide to nutritional status than BMI.
Epilepsy	Anti‐epileptic medications should be reviewed regularly.
	Clinicians should consider withdrawal of anti‐epileptics when women are seizure free for a significant time.
	Video EEG monitoring could provide definitive information about altered neurological states, thereby avoiding the use of anticonvulsant drugs for episodes that are not primarily epileptic (such as vacant spells, respiratory dysrhythmias, episodic laughing, crying or staring).
Scoliosis	A spinal curve should be monitored so that, if corrective surgery may be required, it can be performed early when the chances of a good outcome are greater.
Osteopenia	Calcium and vitamin D supplements are recommended. Periodic screening for low bone density should be considered in all adult women with RTT.
	Vitamin D levels could be checked regularly.
Fractures	Families and carers should have a low index of suspicion of a possible fracture, particularly due to communication difficulties and many women with RTT appearing to have a high pain threshold (Fu et al. 2020)
Mental health	Women with RTT are at risk of depression, which may be underdiagnosed.
	This should be carefully considered if physical causes of somatic symptoms (such as weight loss, reduced mobility, social withdrawal or lack of energy) have been ruled out.
Acute illness	Strenuous efforts should be made to exclude physical causes if a middle‐aged RTT woman is unwell and/or distressed. For some, the explanation for their health problems might not be found and psychological causes should be taken into consideration.
Annual health checks	An annual health check would allow clinicians to ensure that assessments available to women with intellectual disability and women in the general population are available to women with RTT. These should include the option of screening such as breast screening and bowel screening and the management of the menopause.
Everyday life	It is vital that adjustment to adult life away from the parental home is provided by well‐informed staff, and that everyday life is structured and individually planned with adequate stimulation and physical exercise (Hagberg et al. 2001; Bisgaard et al. 2021).
	The capabilities of women with RTT should not be underestimated and opportunities should be provided for repeated practice of their skills (Schönewolf‐Greulich et al. 2017).
	Parental support groups are known to play a crucial role in preventive long‐term care for people with RTT (Smeets et al. 2012).
Prognosis	Clinical assessment of the timing and severity of phenotypic manifestations remain the most important prognostic factors in RTT.

*Note:* Recommendations to support Health and Social Care of Women with RTT.

## 5 Conclusion

This study provides novel information on the natural history of RTT in women over 40 years. They typically have a milder course than usual, with little evidence of slow progression except. Furthermore, demonstrable improvements are often found in features such as hand stereotypies, sleep disturbance, epilepsy, and agitation.

Whilst middle‐aged women with RTT experience the same health issues as other women, they also have particular health needs to consider. Most women lacked regular, systematic clinical review and monitoring for Rett‐specific complications, particularly in the management of epilepsy and nutrition. Annual health checks for people with intellectual disability have been implemented in England and Wales and can improve health and quality of life (Felce et al. 2008). Such health checks would also provide an opportunity to address common health problems in middle‐aged women with RTT.

Given the variability in care for adults with RTT, further studies and standard of care guidelines could help to improve the quality of physical and mental health care available. In the meantime, the authors have proposed some recommendations in Table 9 to support the care of middle‐aged women with RTT. Additional guidance for RTT clinical services is also available (Sloper et al. 2024).

## Author Contributions

Anna Hryniewiecka‐Jaworska collected the data from 2005, adding this to the previous data collected by Dr. Kerr. She documented the consent process for all participants. She extracted the data of all those in BIRSS of > 40 years and drafted the first version of the paper in 2015. Emily Sloper revised the original draft very substantially, before submission, and responded to the reviewers' comments. Angus Clarke was responsible for the ethics committee approval, the oversight of BIRSS and of this research, and also contributed to the interpretation of the data and to revising repeated drafts. Hayley Archer assisted with data interpretation and drafting of the paper. All authors have participated in the Cardiff Rett Syndrome Clinic and approved the final manuscript.

## Ethics Statement

The British Isles Rett Syndrome Survey (BIRSS) was initially maintained with ethics approval from the NHS Multi‐Centre Research Ethics Committee for Scotland and, after the move of BIRSS to Cardiff, the NHS Wales Research Ethics Committee 3. Data were collected and stored with the consent of parents or carers.

## Consent

Consent to participation in BIRSS explicitly included the goal of reporting the collected and anonymised information in medical journal reports.

## Conflicts of Interest

The authors declare no Conflicts of Interest.

## Data Availability

The clinical data analysed for the current study are available from the corresponding author on reasonable request, subject to protection of the identity of the affected individuals.
